# The transcription factor AtGLK1 acts upstream of MYBL2 to genetically regulate sucrose-induced anthocyanin biosynthesis in Arabidopsis

**DOI:** 10.1186/s12870-021-03033-2

**Published:** 2021-05-28

**Authors:** Dongming Zhao, Yuxuan Zheng, lingjun Yang, Ziyu Yao, Jianfeng Cheng, Fang Zhang, Haiyan Jiang, Dong Liu

**Affiliations:** College of Agronomy/Key Laboratory of Crop Physiology, Ecology and Genetic Breeding, Ministry of Education, Jiangxi Agricultural University, Nanchang, 330045 China

**Keywords:** Arabidopsis, AtGLK1, Anthocyanin biosynthesis, MYBL2

## Abstract

**Background:**

The regulation of anthocyanin biosynthesis by various factors including sugars, light and abiotic stresses is mediated by numerous regulatory factors acting at the transcriptional level. Here experimental evidence was provided in order to demonstrate that the nuclear GARP transcription factor AtGLK1 plays an important role in regulating sucrose-induced anthocyanin biosynthesis in Arabidopsis.

**Results:**

The results obtained using real-time quantitative PCR and GUS staining assays revealed that *AtGLK1* was mainly expressed in the green tissues of Arabidopsis seedlings and could be induced by sucrose. The loss-of-function *glk1 glk2* double mutant has lower anthocyanin levels than the *glk2* single mutant, although it has been determined that loss of AtGLK1 alone does not affect anthocyanin accumulation. Overexpression of *AtGLK1* enhances the accumulation of anthocyanin in transgenic Arabidopsis seedlings accompanied by increased expression of anthocyanin biosynthetic and regulatory genes. Moreover, we found that AtGLK1 also participates in plastid-signaling mediated anthocyanin accumulations. Genetic, physiological, and molecular biological approaches demonstrated that AtGLK1 acts upstream of MYBL2, which is a key negative regulator of anthocyanin biosynthesis, to genetically regulate sucrose-induced anthocyanin biosynthesis.

**Conclusion:**

Our results indicated that AtGLK1 positively regulates sucrose-induced anthocyanin biosynthesis in Arabidopsis via MYBL2.

**Supplementary Information:**

The online version contains supplementary material available at 10.1186/s12870-021-03033-2.

## Background

Anthocyanins are a group of plant pigments known to be responsible for the purple coloration of plant parts at particular developmental stages, or under special environmental conditions. The presence of anthocyanin in flowers and fruits is required for attracting pollinators and seed-dispersing animals [1]. Anthocyanins are also an important class of polyphenols which are characterized with remarkable antioxidant activities. Such activities help to protect plants against different abiotic and biotic stress conditions [2–5].

The anthocyanin biosynthetic pathways have been extensively studied in various plant species. The gene encoding enzymes required for the anthocyanin biosynthetic pathways are conserved among different plants [6], and can be grouped into the following two classes [7, 8]. The early biosynthesis genes (EBGs) are involved in the common steps of the different flavonoid subpathways, and mainly include *CHALCONE SYNTHASE* (*CHS*), *CHALCONE ISOMERASE* (*CHI*), and *FLAVANONE 3-HYDROXYLASE* (*F3H*). The late biosynthesis genes (LBGs) primarily include *FLAVONOID 3*'*-HYDROXYLASE* (*F3*'*H*), *DIHYDROFLAVONOL 4-REDUCTASE (DFR)*, *LEUCOANTHOCYANIDIN OXYGENASE* (*LDOX*), *ANTHOCYANIDIN REDUCTASE* (*ANR*), and *UDP-GLUCOSE:FLAVONOID 3-O-GLUCOSYLTRANSFERASE* (*UF3GT*). The expression levels of the aforementioned genes are regulated by positive and negative regulatory transcription factors. For example, it has been determined that the WD-repeat independent MYBs and MYBs/bHLH/WD-repeat complex regulates the expressions of EBGs and LBGs, respectively [9–11]. In Arabidopsis, the transcription factors PIF3 and HY5 positively regulate anthocyanin biosynthesis by directly binding to the promoters of the anthocyanin biosynthetic genes, including *CHS*, *CHI*, *F3H*, *F3′H*, *DFR,* and *LDOX* [12]. In contrast to the positive transcription factors mentioned above, the R3-MYB protein MYBL2 acts as a transcriptional repressor, and negatively regulates the biosynthesis of anthocyanin [13, 14]. Further studies have revealed that MYBL2 inhibits anthocyanin biosynthesis by interacting with TT8 protein to form a transcriptional inhibitory complex which has the ability to bind to the *DFR* promoter and inhibit the transcription of the *DFR* gene [14].

Sugars play essential roles in the growth and development of higher plants, serving as both energy sources and signaling molecules [15]. It has been well established that sucrose is a strong inducer of anthocyanin production in different organs of several plant species [16–19]. The application of exogenous sucrose can significantly increase in the transcript levels of *DFR* and *LDOX* [20, 21]. This sucrose-induced expression of anthocyanin biosynthetic genes may be attributed to the up-regulation expression of positive transcript factors such as *PAP1*, *TT8*, and *GL3* [22]. The sucrose transporters (SUCs) may play an important role in sucrose-induced anthocyanin biosynthesis [18]. It has been found that *AtSUC1* expression levels were higher in sucrose-grown plants when compared with those grown without sucrose. When cultured in sucrose-containing medium, Arabidopsis *suc1* mutants were found to accumulate less anthocyanins. Global expression analyses have revealed reduced expression of many genes important for anthocyanin biosynthesis [23]. Interestingly, *AtSUC1* is preferentially expressed in plant roots, while anthocyanin tends to mainly accumulate in the epidermal layers of the entire abaxial surface, as well as the edges of the adaxial surfaces of the cotyledons [23, 24]. Therefore, it has been indicated that AtSUC1 may play a role in sucrose uptake, rather than acting as a sugar sensor for anthocyanin production [25].

AtGLKs (GOLDEN2-LIKE) are the nuclear GARP transcription factors that have been extensively studied for their roles in regulating chloroplast development [26]. In Arabidopsis, *AtGLK* genes exist as a homologous pairs designated as *AtGLK1* and *AtGLK2*. Although *glk1* and *glk2* single mutants showed no obvious phenotypes throughout the majority of the developmental processes, the *glk1 glk2* double mutant is pale green with a severe reduction in chloroplast thylakoids, suggesting that the AtGLK genes are functionally redundant [26, 27]. Consistent with the rudimentary thylakoid lamellae, the transcript abundance of nuclear genes encoding photosynthesis-related proteins is down-regulated, especially those associated with chlorophyll biosynthesis and PSII [26, 28]. It has also been found that in addition to chloroplast development, AtGLK genes are involved in mediating chloroplast-to-nucleus retrograde signaling in response to the functional states of the chloroplast [29–31]. The *ppi2* (*plastid protein import2*) mutant, which lacks the Toc159 chloroplast preproteins receptor, exhibits repression of photosynthesis-related nuclear genes expression, altered chloroplast morphology, and a severe albino phenotype. Transcript analysis results have revealed that *AtGLK1* expression was significantly down-regulated in the *ppi2* mutant. Furthermore, the expression of some photosynthesis-related genes has been found to be partially restored in transgenic plants overexpressing *AtGLK1* in a *ppi2* background. These findings suggested that AtGLK1 acts as a positive regulator in a chloroplast-to-nucleus signaling pathway that regulates nuclear genes expression in response to the functional status of chloroplasts [29].

In the present research investigation, the identification of AtGLK1 as a positive regulator of sucrose-induced anthocyanin biosynthesis was verified. Our results showed that *AtGLK1* was preferentially expressed in green tissues and it could be induced by exogenous sucrose. Loss-of-function *glk1 glk2* double mutant seedlings were found to have accumulated less anthocyanins in response to sucrose, whereas At*GLK1*-overexpressing Arabidopsis seedlings accumulated more anthocyanins in response to sucrose. Further investigations demonstrated that AtGLK1 acts upstream of MYBL2 to genetically regulate anthocyanin biosynthesis. Therefore, all of the above-mentioned results suggested that AtGLK1 is a key factor which positively regulates sucrose-induced anthocyanin accumulation via MYBL2.

## Results

### *AtGLK1* is a sucrose-inducible gene in Arabidopsis

**Fig. 1:**
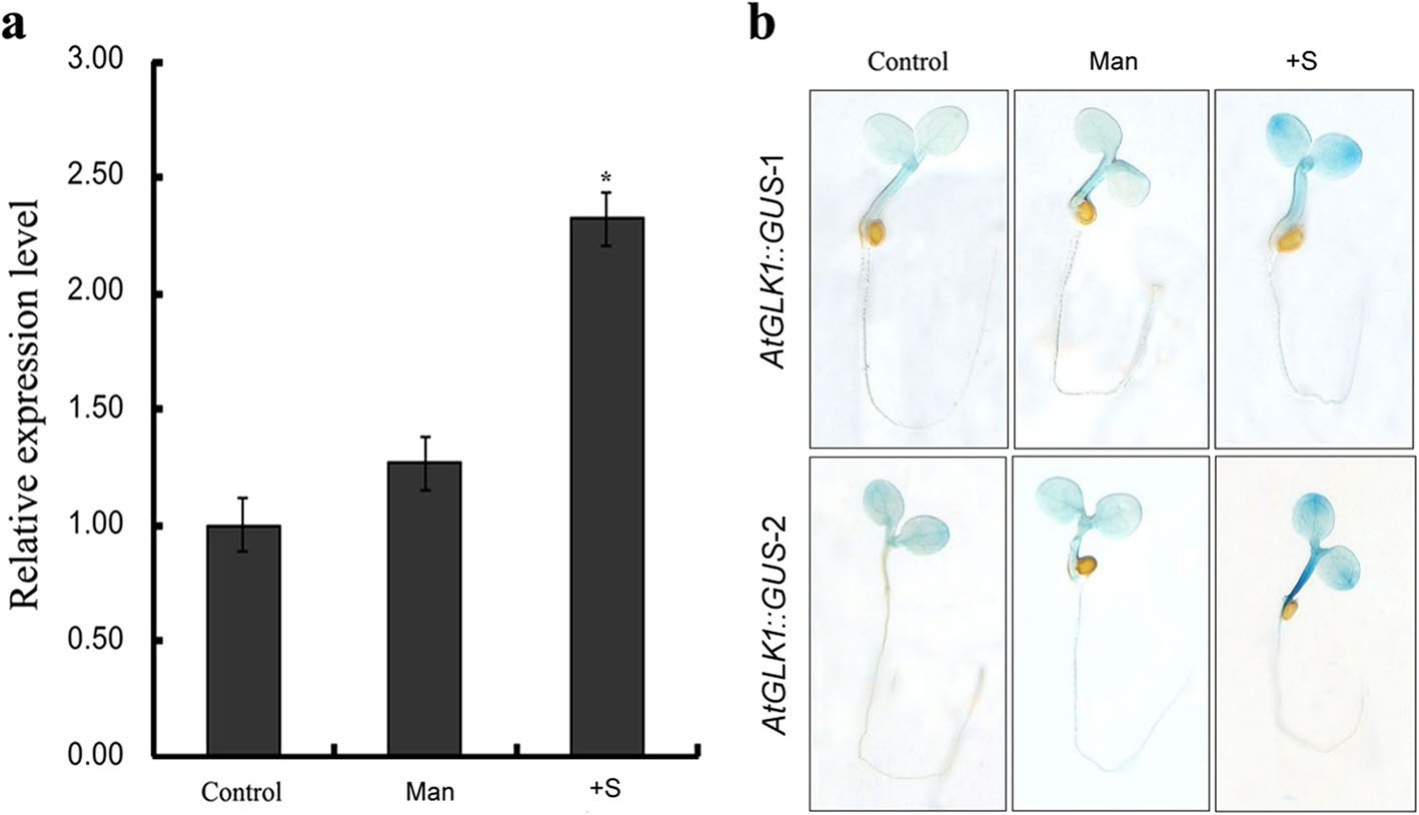
*AtGLK1* is a sucrose-inducible gene in Arabidopsis. **a** Accumulation of *AtGLK1* mRNA under treatment with exogenous sucrose and mannitol. Seeds of the wild-type Arabidopsis (Col) were germinated and grown on 1/2 MS medium without sucrose for 4 days. The 4-day-old seedlings were then transferred to 1/2 MS medium without sucrose (Control), with 58 mM mannitol (Man), or with 2% sucrose (+ S), and each was harvested after 24 h of treatment. The total RNA was extracted and used for real-time PCR. **b** Evaluation of the GUS expression in *AtGLK1::GUS* transgenic Arabidopsis seedlings treated with exogenous sucrose and mannitol. *AtGLK1::GUS* transgenic Arabidopsis seeds from two representative lines (*AtGLK1::GUS*-1 and -2) were germinated and grown on 1/2 MS medium lacking sucrose. On the 4th day after germination, The seedlings were transferred to 1/2 MS medium without sucrose (Control), with 58 mM mannitol (Man), or with 2% sucrose (+ S), and grown for an additional 24 h, and then incubated in a GUS-staining solution. The asterisk indicates statistically significant differences compared with the control (Student’s *t* test: *P < 0.05)

Sugars function as signal molecules to regulate growth, development, and gene expression in higher plants [15]. In order to investigate whether or not the Arabidopsis transcription factor AtGLK1 is involved in responses to sugar signalling, we examined the effects of exogenous sucrose on *AtGLK1* expression levels. In addition, mannitol was included in the experiment as an osmotic control. The results of the real-time quantitative PCR analysis showed that the *AtGLK1* transcript was significantly up-regulated by treatment with 2% sucrose. However, the mannitol treatment did not dramatically increase the *AtGLK1* transcript level (Fig. 1a). In order to further examine the sucrose inductive expression patterns of *AtGLK1*, the *AtGLK1* promoter-controlled GUS activities in response to exogenous sucrose were also analyzed. As shown in Fig. 1b, stronger GUS expression was detected in both the cotyledons and hypocotyls of transgenic *AtGLK1::GUS* Arabidopsis seedlings grown on 1/2 MS medium supplemented with 2% sucrose when compared with the control. Consistent with the qPCR data, it was observed that the expression of *AtGLK1::GUS* was not changed largely after the treatment with mannitol. The sucrose-induced expression of GUS indicated that *AtGLK1* may be involved in plant responses to sugar signaling.

### *AtGLK1 *and* AtGLK2* exhibit functional redundancy in regulating sucrose-induced anthocyanin biosynthesis

**Fig. 2:**
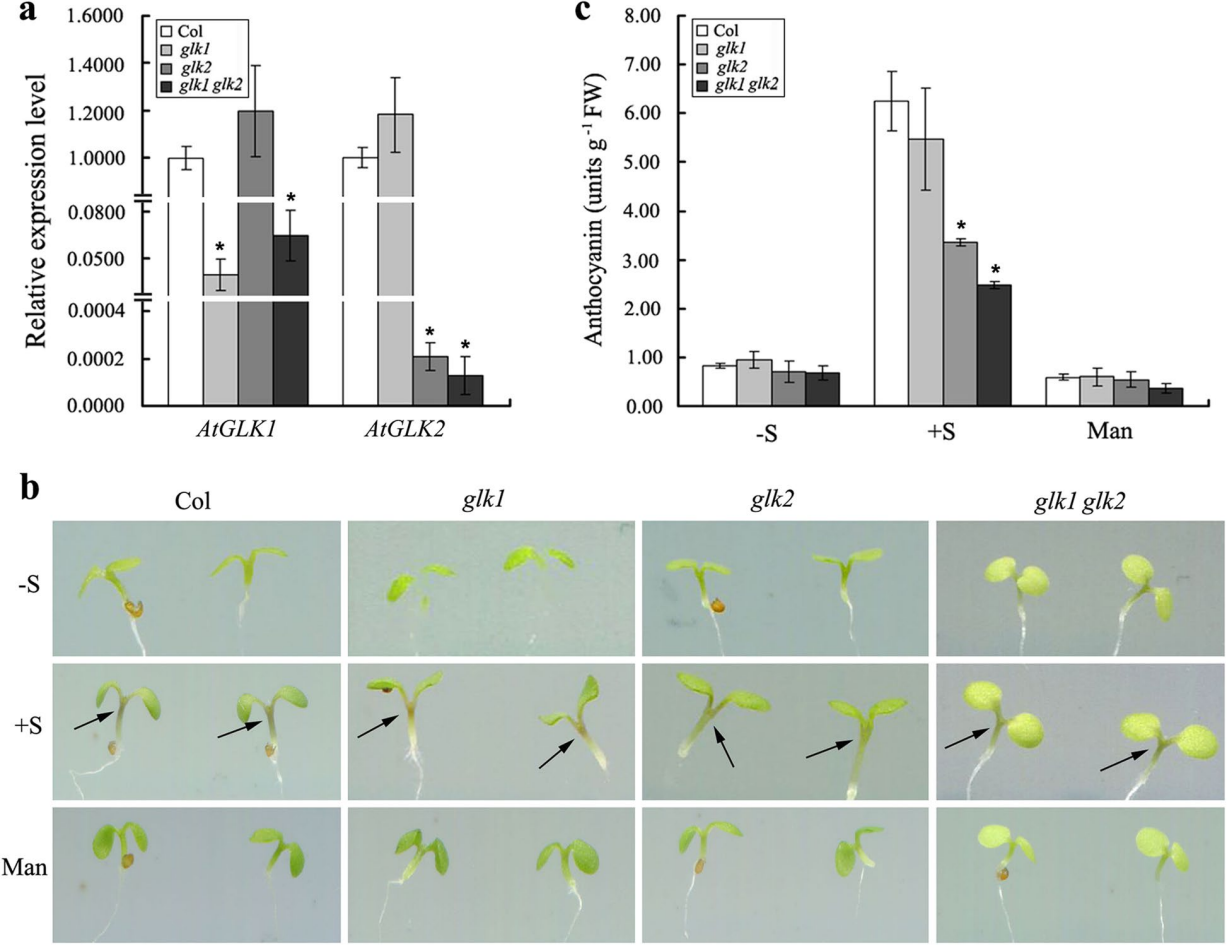
Anthocyanin accumulation in wild-type, single mutants of *glk1* and *glk2,* and the *glk1 glk2* double mutant. **a** Real-time quantitative PCR analysis of the *AtGLK1* and the *AtGLK2* transcript accumulation in the wild type (Col), single mutants of *glk1* and *glk2,* and the *glk1 glk2* double mutant seedlings. The total RNA was isolated from 4-d-old seedlings grown on 1/2 MS medium supplemented with 2% sucrose. **b** Images of representative seedlings of the wild-type (Col), single mutants of *glk1* and *glk2,* and the *glk1 glk2* double mutant grown for 4 days on 1/2 MS medium supplemented without sucrose (-S), with 2% sucrose (+ S), or with 58 mM mannitol (Man), respectively. The black arrows indicate the locations of the anthocyanin accumulation in different genotypic Arabidopsis seedlings. **c** Quantitative measurement of anthocyanins in 4-d-old seedlings (Col, *glk1*, *glk2* and *glk1 glk2*) grown on 1/2 MS medium supplemented without sucrose (-S), with 2% sucrose (+ S), or with 58 mM mannitol (Man), respectively. The asterisks indicate statistically significant differences compared with the corresponding wild-type (Student’s *t* test: *P < 0.05)

It has been well established that sucrose is a strong inducer of anthocyanin production in Arabidopsis [18, 32]. The induction of *AtGLK1* expression by sucrose in Arabidopsis suggested that it may be involved in regulating anthocyanin biosynthesis. In order to confirm this, the single mutants of *glk1* and *glk2* and the *glk1 glk2* double mutant were investigated. These loss-of-function mutants had previously been demonstrated to impact chloroplast development in Arabidopsis [26]. Figure 2a illustrates that the *AtGLK1* transcripts displayed very little accumulation in the *glk1* mutant. However, they were present at normal levels in the *glk2* mutant. Similarly, the *AtGLK2* transcripts were observed to be very low in the *glk2* mutant but were accumulated to normal levels in the *glk1* mutant. The transcript levels of both the *AtGLK1* and *AtGLK2* genes were very low in the *glk1 glk2* double mutant. Seeds of both the wild type and the *glk* mutants (*glk1*, *glk2,* and *glk1 glk2*) were germinated and grown vertically on 1/2 MS medium supplemented with 2% sucrose for 4 days following stratification. It was observed that the anthocyanin accumulations in the *glk2* single mutant and the *glk1 glk2* double mutant seedlings were significantly decreased in the upper part of hypocotyls, when compared with that of the corresponding wild-type seedlings. However, when the seedlings were germinated and grown on 1/2 MS medium without sucrose or with 58 mM mannitol, no significant differences could be observed among the wild-type, the single mutants of *glk1* and *glk2*, and the *glk1 glk2* double mutant (Fig. 2b). Quantitative analysis showed that the anthocyanin contents of seedlings grown in the absence of sucrose were fairly low and there were no significant differences observed between the *glk* mutants (*glk1*, *glk2*, and *glk1 glk2*) and the wild-type seedlings. However, there were marked inductions of anthocyanin accumulations in both the wild-type and *glk* mutants (*glk1*, *glk2*, and *glk1 glk2*) in the presence of sucrose. Although no significant differences were observed in the anthocyanin contents between the wild-type and *glk1* mutant, the anthocyanin contents of *glk2* single mutant and *glk1 glk2* double mutant were found to be significantly lower than those of the wild-type seedlings. Furthermore, the *glk1 glk2* double mutant was observed to be more defective in anthocyanin accumulation when compared with the *glk2* single mutant. In order to determine if the differences in the anthocyanin accumulation levels in the wild-type and *glk* mutants (*glk1*, *glk2*, and *glk1 glk2*) seedlings were due to osmotic effects, the seedlings were also grown on equimolar concentrations of mannitol (58 mM = 2%), and the anthocyanin contents were assayed. The mannitol failed to induce anthocyanin accumulations in either the wild-type or the *glk* mutants (*glk1*, *glk2*, and *glk1 glk2*) seedlings, which suggested that the sucrose-induced anthocyanin accumulations could not be regarded as an osmotic effect (Fig. 2c).

### Overexpression of* AtGLK1* enhances sucrose-induced anthocyanin accumulation in Arabidopsis

**Fig. 3:**
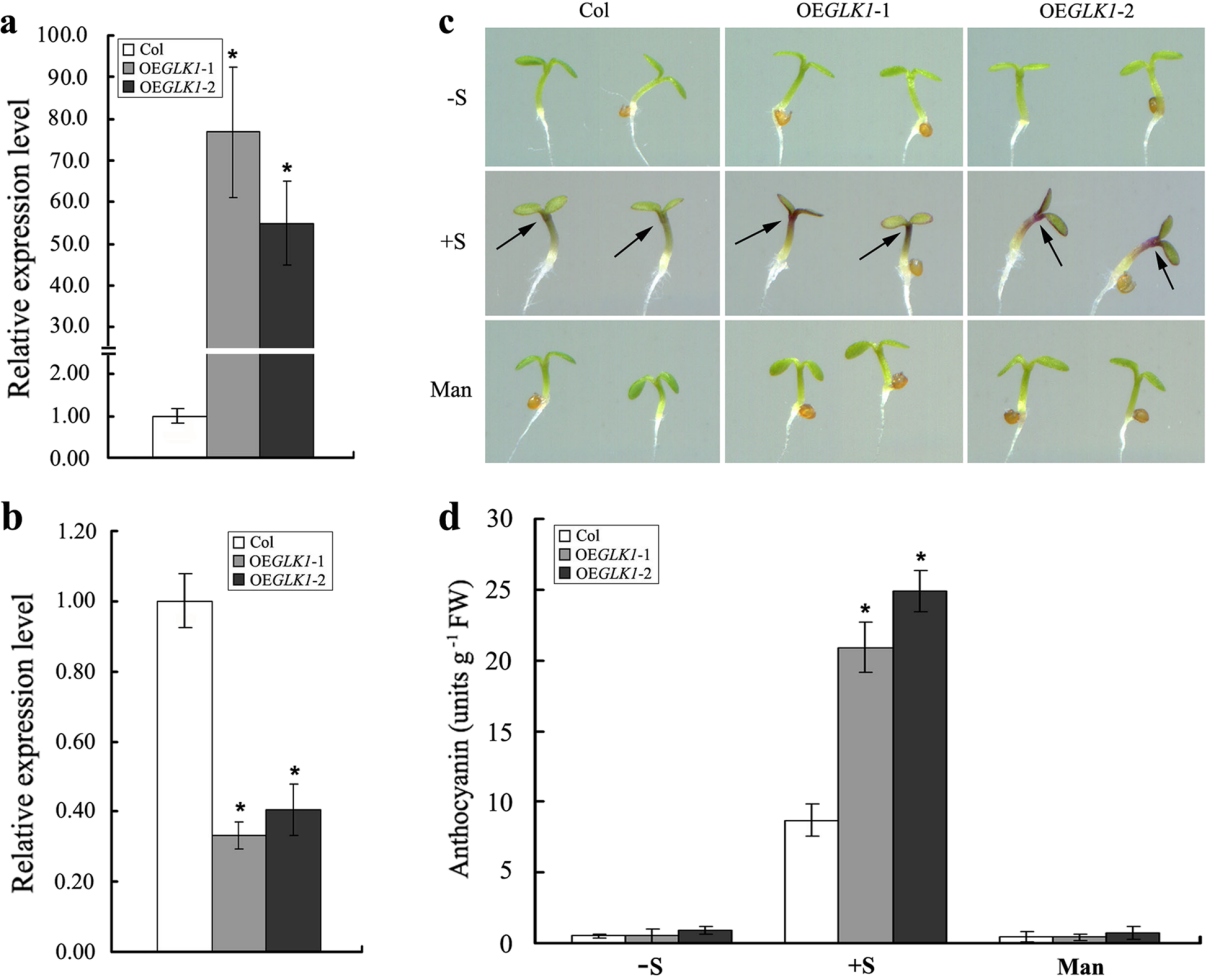
Anthocyanin accumulation in the wild-type and *AtGLK1*-overexpressing seedlings. **a** Expression analysis of *AtGLK1* and *AtGLK2* **b** in the wild-type (Col) and *35S::AtGLK1* transgenic lines (OE*GLK1*-1 and OE*GLK1*-2). Total RNA extracted from the 4-d-old wild-type and *35S::AtGLK1* transgenic seedlings was used for real-time quantitative PCR analyses. **c** Images of representative seedlings of the wild-type and *35S::AtGLK1* transgenic lines grown for 4 days on 1/2 MS medium supplemented without sucrose (-S), with 2% sucrose (+ S), or with 58 mM mannitol (Man), respectively. The black arrows indicate the locations of anthocyanin accumulation in different genotypic Arabidopsis seedlings. **d** Quantitative measurement of anthocyanins in the 4-day-old seedlings (Col, OE*GLK1*-1 and OE*GLK1*-2) grown on 1/2 MS medium supplemented without sucrose (-S), with 2% sucrose (+ S), or with 58 mM mannitol (Man), respectively. The asterisks indicate statistically significant differences compared with the corresponding wild-type (Student’s *t* test: *P < 0.05)

To investigate whether or not the accumulation of anthocyanin was affected in *AtGLK1*-overexpressing lines, the *AtGLK1* gene, driven by *CaMV 35S* promoter, was introduced into Arabidopsis. 9 independent *35S::AtGLK1* transgenic lines were obtained on a selection 1/2 MS medium with 50 μg ml^−1^ kanamycin. Through kanamycin-resistance assay and PCR analysis (data not shown), the homozygous transgenic progeny lines (T3 to T4 generations) were selected for further examination. The expression levels of two representative independent transgenic lines (OE*GLK1*-1 and OE*GLK1*-2) were examined using real-time quantitative PCR analysis with gene-specific primers. As expected, the transgenic lines OE*GLK1*-1 and OE*GLK1*-2 were found to have higher relative expression levels of *AtGLK1* when compared with the wild type (Fig. 3a). We also detected the expression of *AtGLK2*, a homologous gene to *AtGLK1*, in the wild-type and *35S::AtGLK1* transgenic plants. It was interesting to note that the expression of *AtGLK2* was found to be significantly impaired in the *AtGLK1*-overexpressing seedlings, when compared with the corresponding wild-type plants (Fig. 3b).

When grown on 1/2 MS medium in the absence of sucrose, the anthocyanin accumulation in the *AtGLK1*-overexpressing seedlings was indistinguishable from that in wild-type seedlings, a result similar to that observed in the seedlings grown on 1/2 MS medium in the presence of 58 mM mannitol. However, we observed an obvious difference in the anthocyanin pigmentation intensity in the upper part of the hypocotyls of these seedlings in the presence of sucrose. In comparison with wild-type seedlings, clear increases in the level of purple anthocyanin were observed in both of the selected *AtGLK1* overexpression lines (Fig. 3c). Quantification of the anthocyanin level validated the phenotypic observations and confirmed the higher anthocyanin levels in both the selected *AtGLK1* overexpression lines when compared with the wild-type seedlings (Fig. 3d). Taken together, the data obtained in this study revealed a positive correlation between the *AtGLK1* expression and anthocyanin accumulation in the Arabidopsis seedlings.

### Expression of the structural and regulatory genes of the anthocyanin biosynthetic pathway

**Fig. 4:**
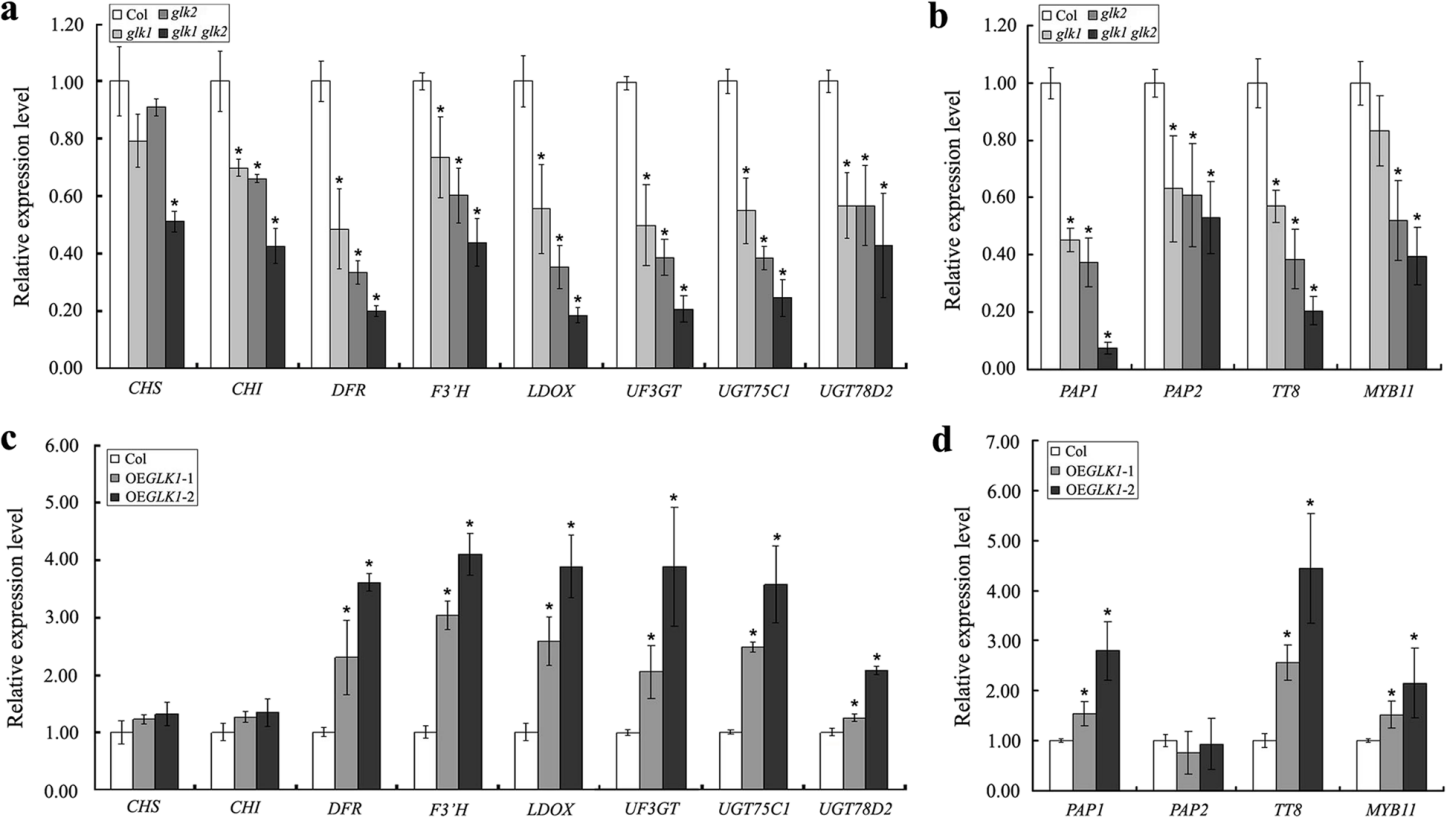
Expression levels of the structural and regulatory genes of the anthocyanin biosynthetic pathway. **a**-**b** Transcript levels of the structural (*CHS*, *CHI*, *DFR*, *F3*'*H*, *LDOX*, *UF3GT*, *UGT75C1*, and *UGT75C2*) and regulatory (*PAP1*, *PAP2*, *TT8*, and *MYB11*) genes involved in anthocyanin biosynthesis in the *glk* mutants (*glk1*, *glk2*, and *glk1 glk2*) and *AtGLK1*-overexpressing seedlings (**c**-**d**). Total RNA extracted from the 4-day-old wild-type (Col), *glk* mutants and *AtGLK1*-overexpressing (OE*GLK1*-1 and OE*GLK1*-2) seedlings was used for real-time quantitative PCR analyses. The asterisks indicate statistically significant differences compared with the corresponding wild-type (Student’s *t* test: *P < 0.05)

The results described above indicated that AtGLK1 is involved in the regulation of anthocyanin synthesis. Therefore, in order to more clearly understand the molecular basis of the changes in anthocyanin levels, we first examined the expression of the early biosynthetic genes *CHALCONE SYNTHASE* (*CHS*) and *CHALCONE ISOMERASE* (*CHI*) using reverse transcription followed by real-time quantitative PCR. As detailed in Fig. 4, The transcript levels of *CHS* had not dramatically changed in the single mutants of *glk1* and *glk2.* However, it was found that the *CHI* transcript levels were clearly decreased in the two mutants. In addition, when compared with the wild type, it was observed that expression levels of the *CHS* and *CHI* were not greatly changed in the *AtGLK1* overexpression lines. However, the expression of both genes was majorly decreased in the *glk1 glk2* double mutant. We then monitored the expression levels of the following late biosynthetic genes *DIHYDROFLAVONOL 4-REDUCTASE* (*DFR*), *FLAVONOID 3′ HYDROXYLASE* (*F3′H*), *LEUCOANTHOCYANIDIN OXYGENASE* (*LDOX*), *UDP-GLUCOSE:FLAVONOID 3-O-GLUCOSYL TRANSFERASE* (*UF3GT*), *UDP-GLUCOSYL TRANSFERASE 75C1* (*UGT75C1*), and* UDP-GLUCOSYL TRANSFERASE 78D2* (*UGT78D2*). The late biosynthetic genes showed the same expression patterns, in which the transcript levels of the genes were lower in the *glk* mutants (*glk1*, *glk2*, and *glk1 glk2*) than in the wild type but higher in the *AtGLK1*-overexpressing lines. Subsequently, the expression levels of several regulatory genes in the anthocyanin biosynthetic pathways were further examined, including *PRODUCTION OF ANTHOCYANIN PIGMENT 1* (*PAP1*), *PRODUCTION OF ANTHOCYANIN PIGMENT 2* (*PAP2*), *TRANSPARENT TESTA 8* (*TT8*)*,* and *MYB11*. As expected, the *PAP1*, *TT8,* and *MYB11* expressions were found to be consistently and substantially higher in the *AtGLK1*-overexpressing lines when compared with the wild type but lower in the *glk* mutants (*glk1*, *glk2*, and *glk1 glk2*). However, there were no significant differences observed in the gene expression levels of the *PAP2* between the wild type and *AtGLK1*-overexpressing transgenic lines, while its expression was dramatically decreased in the *glk* mutants (Fig. 4). In summary, the results obtained in this study suggested that AtGLK1 positively regulates anthocyanin accumulations in *Arabidopsis* seedlings through modulating the expression levels of structural and regulatory anthocyanin biosynthetic genes.

### AtGLK1 participates in the plastid retrograde signal-mediated anthocyanin accumulation in Arabidopsis

**Fig. 5:**
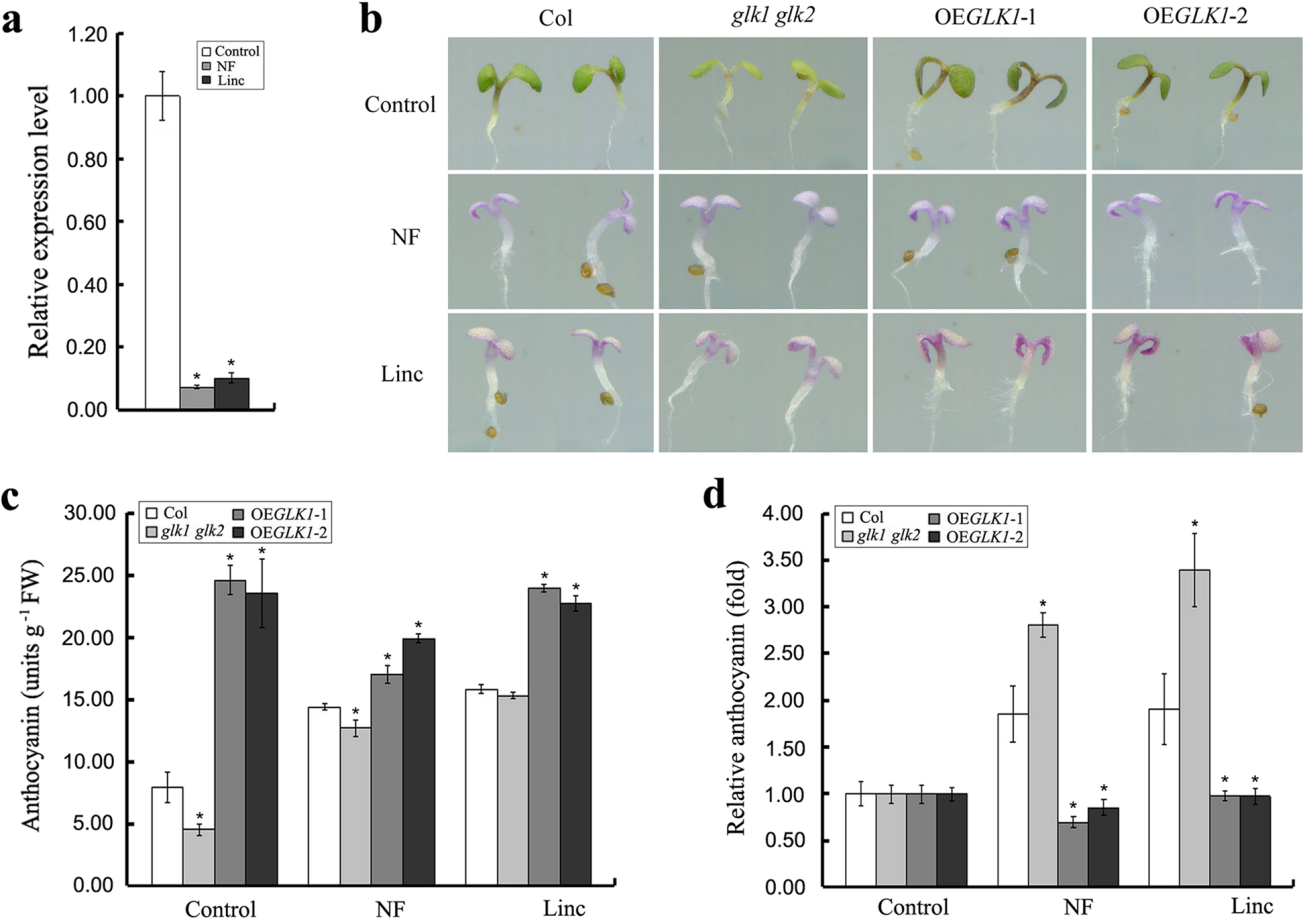
AtGLK1 participates in the plastid retrograde signal-mediated anthocyanin accumulation in Arabidopsis. **a** Real-time quantitative PCR analysis of *AtGLK1* transcript accumulation in wild-type seedlings grown on 1/2 MS medium supplemented without (Control) or with 5 μM norflurazon (NF) or with 0.5 mM lincomycin (Linc). **b** Images of representative seedlings of the wild-type (Col), *glk1 glk2* double mutant, and *AtGLK1*-overexpressing (OE*GLK1*-1 and OE*GLK1*-2) seedlings grown for 4 days on 1/2 MS medium supplemented without (Control) or with 5 μM norflurazon (NF) or with 0.5 mM lincomycin (Linc). **c** Absolute anthocyanin contents and relative anthocyanin contents **d** of the wild-type, *glk1 glk2* double mutant, and *AtGLK1*-overexpressing seedlings grown on 1/2 MS medium for 4 days. The asterisks indicate statistically significant differences compared with the corresponding wild-type (Student’s *t* test: *P < 0.05)

Since AtGLK1 is an important component of the plastid retrograde signal pathway [29], whether AtGLK1 participates in the plastid retrograde signal-mediated anthocyanin accumulation was further investigated. Therefore, wild-type seedlings were treated with norflurazon (NF) or lincomycin (Linc), which are two drugs known to activate retrograde signaling by inhibiting chloroplast biogenesis [33, 34]. The results of the real-time quantitative PCR analysis showed that the *AtGLK1* gene was strongly down-regulated by the NF and Linc treatments at the transcription level (Fig. 5a). Next, NF or Linc were used to treat wild-type Arabidopsis, *glk1 glk2* double mutant, and *AtGLK1*-overexpressing seedlings, and the anthocyanin contents of these samples were then determined. The results are shown in Fig. 5b-c and Fig. S1. For the wild-type seedlings, both NF and Linc were determined to have significantly induced anthocyanin accumulation and the expression of anthocyanin biosynthetic and regulatory genes. In the control group, the anthocyanin accumulation was observed to be lower in the *glk1 glk2* double mutant but higher in *AtGLK1* overexpression lines when compared with the wild type. Treatments with NF and Linc significantly induced anthocyanin accumulation in wild-type and *glk1 glk2* double mutant seedlings. However, no significant inductive effects were observed in either of the *AtGLK1*-overexpressing lines (Fig. 5c). Since the absolute anthocyanin contents in the untreated control seedlings were found to have significant differences among all of the genotypes, the relative anthocyanin contents (fold of the anthocyanin contents in the treatments to the mean of the control) were calculated. The results of the statistical analysis revealed significant differences in the levels of relative anthocyanin between the *glk1 glk2* double mutant and the *AtGLK1*-overexpressing seedlings, the inductive effects of both the NF and Linc treatments on the anthocyanin accumulations were found to be further enhanced in the *glk1 glk2* double mutant but were significantly decreased in the *AtGLK1*-overexpressing lines (Fig. 5d). These findings suggested that AtGLK1 participates in plastid retrograde signal-mediated anthocyanin accumulation in Arabidopsis.

### AtGLK1 acts upstream of MYBL2 to genetically regulate anthocyanin accumulation in Arabidopsis

**Fig. 6:**
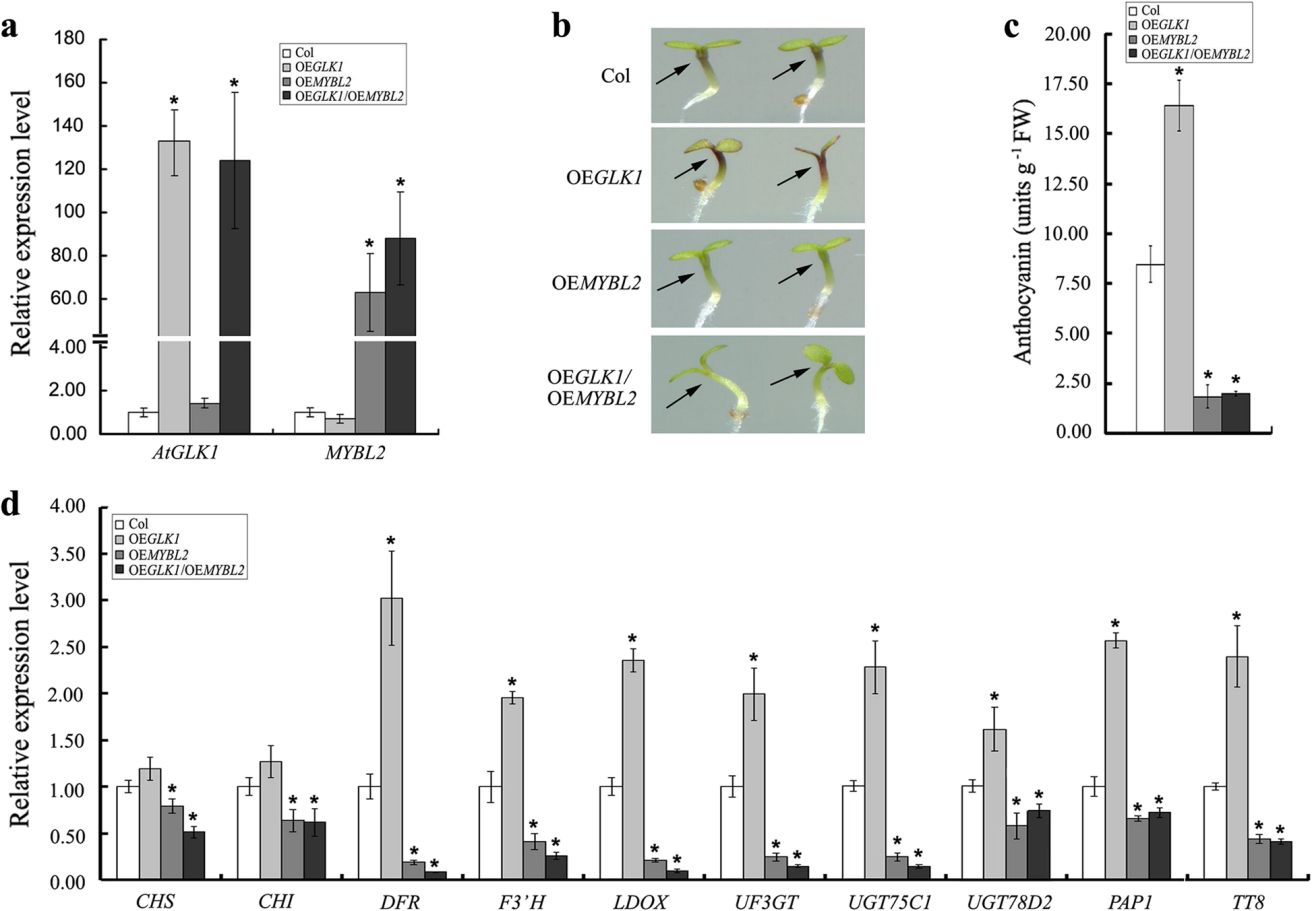
AtGLK1 acts upstream of MYBL2 to genetically regulate anthocyanin accumulation in Arabidopsis. **a** Expression analysis of *AtGLK1* and *MYBL2* in the wild-type (Col), *AtGLK1*-overexpressing (OE*GLK1*), *MYBL2*-overexpressing (OE*MYBL2*), and *35S::MYBL2*/*OEGLK1* (*OEGLK1/OEMYBL2*) double overexpressing plants. **b** Images of representative seedlings and anthocyanin contents **c** of wild-type, *AtGLK1*-overexpressing, *MYBL2*-overexpressing, and *35S::MYBL2*/*OEGLK1* double overexpressing lines. **d** Transcript levels of structural (*CHS*, *CHI*, *DFR*, *F3*'*H*, *LDOX*, *UF3GT*, *UGT75C1*, and *UGT75C2*) and regulatory (*PAP1* and *TT8*) genes involved in anthocyanin biosynthesis in wild-type, *AtGLK1*-overexpressing, *MYBL2*-overexpressing, and *35S::MYBL2*/*OEGLK1* double overexpressing plants. Total RNA extracted from the different genotypic seedlings grown on 1/2 MS medium for 4 days. The asterisks indicate statistically significant differences compared with the corresponding wild-type (Student’s *t* test: *P < 0.05)

It has been previously reported that MYBL2 acts as a transcriptional repressor and negatively regulates the biosynthesis of anthocyanin in Arabidopsis [13, 14]. In the *MYBL2* knockout line (*mybl2*), the expression of the anthocyanin biosynthetic and regulatory genes was enhanced and resulted in the ectopic accumulation of anthocyanin, while ectopic expression of *MYBL2* or of a chimeric repressor that is a dominant negative form of MYBL2 suppressed the expression of anthocyanin biosynthetic and regulatory genes, and the biosynthesis of anthocyanin [13, 14]. To determine the genetic relationship between AtGLK1 and MYBL2, the *35S::MYBL2* (*OEMYBL2*) was crossed with the *AtGLK1* overexpression line (*OEGLK1*), and the double overexpressing line *35S::MYBL2*/*OEGLK1* (OE* GLK1/OEMYBL2*) was obtained (Fig. 6a). Our results showed that the overexpression of *MYBL2* significantly suppressed the anthocyanin biosynthesis of *AtGLK1*-overexpressing seedlings, which indicated that MYBL2 was epistatic to *AtGLK1* in anthocyanin biosynthesis (Fig. 6b-c). Consistency was observed in the transcript levels of the anthocyanin biosynthetic (*DFR*, *F3′H*, *LDOX*, *UF3GT*, *UGT75C1*, and *UGT78D2*) and regulatory (*PAP1* and *TT8*) genes, which were dramatically up-regulated in *AtGLK1*-overexpressing seedlings, all were down-regulated when the *MYBL2* was overexpressed in the *35S::MYBL2*/*OEGLK1* (OE* GLK1/OEMYBL2*) double overexpressing line (Fig. 6d). Therefore, these results indicated that AtGLK1 acts upstream of MYBL2 to genetically regulate anthocyanin accumulation in Arabidopsis.

## Discussion

In higher plants, the regulation of anthocyanin biosynthesis by various transcription factors [9–14, 32, 35]. The GLK transcription factors were originally identified in maize, and were subsequently found in Arabidopsis, maize, rice, tomato, and the moss *Physcomitrella patens* [26, 27, 36–39]. GLK transcription factors belong to the GARP transcription activator family, and the protein sequences are highly conserved among different species, with Myb-like DNA-binding domain and the C-terminal box [26, 39, 40]. In Arabidopsis, *AtGLK* genes exist as a pair of homologous genes, *AtGLK1* and *AtGLK2*. The previous studies found that AtGLKs mainly regulate the chloroplast development in higher plants [26–28]. In recent years, more and more studies have shown that AtGLKs play important roles not only in responding to biotic and abiotic stresses, but also in regulating leaf senescence [41–45]. The current study found that AtGLKs have an important function in regulating the accumulation of anthocyanins in Arabidopsis.

Anthocyanins are water-soluble, vacuolar pigments in plants that belong to the family of flavonoid compounds [46]. Since sucrose is a strong inducer of flavonoid biosynthesis and is known to induce anthocyanin accumulation in a variety of plant species [16–18], we analyzed the expression patterns of *AtGLK1* in response to exogenous sucrose treatment. Real-time quantitative PCR analyses revealed that the mRNA accumulation of *AtGLK1* was significantly promoted by sucrose (Fig. 1a). The increased *AtGLK1* transcript level in response to sucrose appeared to originate from its promoter activities since it was observed that exogenous sucrose treatments significantly increased GUS expression in the cotyledons and hypocotyl of *AtGLK1::GUS* transgenic seedlings (Fig. 1b). Such an expression pattern suggested that AtGLK1 may be involved in sucrose-induced anthocyanin accumulation during the early stages of Arabidopsis development.

Through the phenotypic, physiological, and molecular analyses conducted in this work, strong positive correlations were identified between *AtGLK1* expression and anthocyanin accumulation to sucrose treatment. First, the loss-of-function *glk1 glk2* double mutant was found to have lower anthocyanin levels than the *glk2* single mutant, although loss of AtGLK1 alone had not affected the anthocyanin accumulation (Fig. 2). The absence of an anthocyanin-less phenotype for the *glk1* mutant may have been due to functional redundancy or compensation between the AtGLK1 and AtGLK2. Similarly, the AtGLK1 and AtGLK2 have been shown to be functionally redundant in the regulation of chloroplast development [26, 27]. During the early developmental stage of Arabidopsis seedlings, single *glk* mutants (*glk1* and *glk2*) largely resemble wild-type, only the *glk1 glk2* double mutant showed a chloroplast-defective phenotype, suggesting that the each of two *AtGLK* genes acts redundantly to direct monomorphic chloroplast development [26]. The *AtGLK* genes were found to exhibit partial redundancy since there was an anthocyanin-less phenotype specific to the *glk2* mutant allele, but no phenotype specific to the *glk1* allele (Fig. 2). The following two aspects of the experimental data may have reflected the fact that the two genes had different expression levels rather than different functions. On the one hand, overexpression of *AtGLK1* significantly enhanced anthocyanin accumulation in the *35S::AtGLK1* transgenic Arabidopsis seedlings, even though the expression of *AtGLK2* was dramatically impaired (Fig. 3). On the other hand, real-time quantitative PCR results showed that the mRNA accumulation of *AtGLK1* was significantly lower than that of *AtGLK2* in the wild-type Arabidpsis seedlings (Fig. S2). Second, when overexpressed in Arabidopsis, the *35S::AtGLK1* transgenic seedlings displayed enhanced anthocyanin accumulation (Fig. 3). We also detected the expression of *AtGLK2* in the wild-type and *35S::AtGLK1* transgenic seedlings. It was interesting to find that the expression of *AtGLK2* was significantly impaired in the *AtGLK1*-overexpressing plants when compared with the corresponding wild-type plants (Fig. 3b). There were two possible explanations. The first explanation was that the AtGLK1 has an additional function of regulating *AtGLK2* expression. The second explanation is that the decreased transcription of the *AtGLK2* in the *AtGLK1*-overexpressing plants were most likely for the purpose of maintaining a constant total mRNA amount of *AtGLKs* via expressional reprogramming between the two homologous genes. Third, We found that *glk* mutants (*glk1*, *glk2* and *glk1 glk2*) seedlings had accumulated lower transcript levels of *DFR*, *F3*'*H*, *LDOX*, *UF3GT*, *UGT75C1*, and *UGT75C2*, which are known to be involved in the late step of anthocyanin biosynthesis, while the *AtGLK1*-overexpressing seedlings showed higher transcript levels than those observed in the wild-type seedlings (Fig. 4). In contrast, the transcript levels of the early biosynthesis genes, such as *CHS* and *CHI*, were not observed to be greatly altered in the *AtGLK1*-overexpressing plants (Fig. 4c). Another potential target of AtGLK1 action could be *PAP1*, which has been shown to trigger the activation of expression of late anthocyanin biosynthesis genes [18, 47]. PAP1 is an R2R3 MYB-type transcription factor that is capable of mediating ectopic activation of an array of genes involved in anthocyanin biosynthesis in several plant species, including Arabidopsis, tobacco, petunia and rose [47–50]. Indeed, our study found that the transcript level of *PAP1* was lower in the *glk* mutants (*glk1*, *glk2*, and *glk1 glk2*) seedlings, but significantly higher in *AtGLK1*-overexpressing seedlings, when compared with the corresponding wild-type plants (Fig. 4). It therefore appeared that the AtGLK1 regulates sucrose-induced anthocyanin accumulation mainly through influencing the expression of late anthocyanin biosynthesis genes. Therefore, based on the results mentioned above, our study considered that AtGLK1 is potentially a positive regulator of anthocyanin accumulation in Arabidopsis.

The intracellular signaling from the chloroplast to the nucleus is referred to as plastid retrograde signaling. These signaling processes play essential roles in coordinating the expression of nuclear and plastid-encoded genes [51]. In the present study, it was found that norflurazon and lincomycin (two drugs known to block chloroplast biogenesis via different mechanisms), which induce retrograde signaling [33, 34], were found to enhance the anthocyanin accumulation of sucrose-treated Arabidopsis seedlings (Fig. 5; Fig. S1). These findings suggested that the anthocyanin biosynthesis is positively regulated by plastid retrograde signaling. If the positive signals from dysfunctional chloroplasts are transmitted exclusively via AtGLK1, then these signals should be abrogated in *glk1 glk2* double mutants. However, the effects of norflurazon and lincomycin on the sucrose-induced anthocyanin accumulation were observed to be greater in the *glk1 glk2* double mutants, but lower in *AtGLK1*-overexpressing seedlings, when compared with wild-type seedlings (Fig. 5c-d). These observations suggested the possibility that AtGLK1 acts as a negative regulator in plastid retrograde signal-mediated anthocyanin accumulation. Consistent with this speculation, the results of the real-time quantitative PCR analysis showed that the *AtGLK1* had been strongly down-regulated by the norflurazon and lincomycin treatments at the transcription level (Fig. 5a). Despite this, further studies will be needed in order to unravel the detailed molecular mechanisms of AtGLK1-mediated plastid retrograde signaling pathways which regulate anthocyanin accumulation.

MYBL2 is a negative regulator of anthocyanin biosynthesis. The analyses of the expression patterns of the *mybl2* mutant, or transgenic plants overexpressing *MYBL2,* have demonstrated that MYBL2 regulates the expression of anthocyanin biosynthesis-related genes [13, 14]. Similar expression patterns were observed in the structural and regulatory genes in the anthocyanin biosynthetic pathways in the *AtGLK1*-overexpressing plants and the *glk1 glk2* double mutant in this study (Fig. 4), which raised the possibility that AtGLK1 regulates anthocyanin biosynthesis by modulating *MYBL2* expression. However, the *MYBL2* transcript levels showed no obvious changes in either the *glk1 glk2* double mutant or *AtGLK1*-overexpressing plants when compared with the wild-type (data not shown). Therefore, it was hypothesized that AtGLK1 may regulate *MYBL2* expression at the post-transcriptional level. To determine the genetic relationship between AtGLK1 and MYBL2, we generated transgenic lines overexpressing *MYBL2* in *AtGLK1*-overexpressing plants. The results indicated that the overexpression of *MYBL2* completely complemented the anthocyanin overaccumulation phenotype in the *AtGLK1*-overexpressing seedlings (Fig. 6b-c), which suggested that MYBL2 is epistatic to AtGLK1 in anthocyanin biosynthesis. Also, consistency was found in the transcript levels of the anthocyanin biosynthetic (*DFR*, *F3*'*H*, *LDOX*, *UF3GT*, *UGT75C1*, and *UGT75C2*) and regulatory (*PAP1* and *TT8*) genes, which were up-regulated in the *AtGLK1*-overexpressing seedlings, and all down-regulated when *MYBL2* was overexpressed (Fig. 6d).

## Conclusion

In summary, the results obtained in this study indicated that in addition to regulating chloroplast development [26], abiotic and biotic stress responses [41, 42, 44, 45], and leaf senescence [43], AtGLK1 positively regulates sucrose-induced anthocyanin biosynthesis in Arabidopsis. Furthermore, it was determined that MYBL2 plays an important genetical role in the downstream of AtGLK1. It is believed that future research will clarify the exact molecular mechanisms of the AtGLK1-mediated plastid signaling pathways which regulate anthocyanin accumulation.

## Methods

### Plant material and growth conditions

The wild type and mutant lines of *Arabidopsis thaliana* were all in the Columbia ecotype (Col-0). Transfer DNA insertion mutants *glk1* (CS9805), *glk2* (CS9806), and *glk1 glk2* (CS9807) were obtained from the Arabidopsis Biological Resource Center (ABRC), and the transgenic Arabidopsis plants overexpressing both the *AtGLK1* and *MYBL2* (OE*GLK1*/OE*MYBL2*) were produced by crossing transgenic homozygous lines overexpressing *AtGLK1* and *MYBL2*. Following 3 days of stratification in the dark at 4 °C, the surface-sterilized seeds were germinated on 1/2 MS medium [0.8% (w/v) agar, 2% (w/v) sucrose, pH 5.8] at 22 °C with a 16-h-light/8-h-dark cycle unless otherwise stated. All phenotypic characterization experiments were conducted on multiple biological samples and repeated at least 3 times.

To examine the effects of norflurazon (NF) and lincomycin (Linc) on anthocyanin biosynthesis, the sterilized and cold-treated seeds were germinated and grown vertically on 1/2 MS medium without (Mock) or with 5 μM NF or with 0.5 mM Linc for 4 days (under continuous light conditions). The 4-day-old seedlings were then harvested for anthocyanin measurement.

### Verification of *dSpm* insertions in *glk* mutants

The *dSpm* insertions in *glk1* and *glk2* mutants were confirmed by PCR using *dSpm*-specific primers, with spm5 for *glk1* and spm8 for *glk2*; and *AtGLK* genes-specific primers, with 2bgs2 for *glk1*, and ara4 for *glk2*. PCR genotyping primers are listed in Table S1 and the results of PCR genotyping of the mutants are shown in Fig. S1.

### Constructs and plant transformation

To construct the *AtGLK1::GUS* fusion gene, a 1,702-bp DNA fragment upstream of the ATG start codon of the *AtGLK1* gene (At2g20570) was amplified from *Arabidopsis thaliana* genomic DNA by PCR. The pair of primers used in the PCR was *PGLK1-F* and *PGLK1-R* (*Bam*H I and *Nco* I sites were introduced). The specific PCR fragment was then inserted into binary vector pCAMBIA 1301 between *Bam*H I and *Nco* I sites, replacing the *CaMV 35S* promoter, to create the recombinant transcription unit *AtGLK1::GUS*. For the construction of *35S::AtGLK1* unit, the full-length coding sequence (CDS) corresponding to the *AtGLK1* gene locus was cloned by using RT-PCR from *Arabidopsis thaliana*. The pair of primers used in the PCR was *OEGLK1-F* and *OEGLK1-R* (*Xba* I and *Sac* I sites were introduced). The specific PCR fragment was then inserted into binary vector PBI 121 between *Xba* I and *Sac* I sites, replacing the *GUS* gene, to create the recombinant transcription unit *35S::AtGLK1.* For the construction of *35S::MYBL2* unit, the full-length coding sequence (CDS) corresponding to the *MYBL2* gene (At1g71030) locus was cloned by using RT-PCR from *Arabidopsis thaliana*. The pair of primers used in the PCR was *OEMYBL2-F* and *OEMYBL2-R* (*Nco* I and *Bst*E II sites were introduced). The specific PCR fragment was then inserted into binary vector pCAMBIA 1301 between *Nco* I and *Bst*E II sites, replacing the *GUS* gene, to create the recombinant transcription unit *35S::MYBL2.* All primers used are listed in Table S1.

The recombinant plasmids were then introduced into *Agrobacterium tumefaciens* strain GV3101 and transformed into wild-type Arabidopsis (Col-0) using the floral dip method [52]. The transformants were then screened on 1/2 MS medium containing 50 μg ml^−1^ Kanamycin (*35S::AtGLK1*) or 50 μg ml^−1^ hygromycin (*AtGLK1::GUS* and *35S::MYBL2*).

### RNA extraction, cDNA synthesis, and gene expression analysis

RNA extraction and cDNA synthesis were performed according to the method reported in the previous work [53]. For real-time quantitative PCR analysis, the reaction was performed using SYBR Green Perfect mix (TaKaRa, Dalian, China) on a CFX96 (Bio-Rad), following the manufacturer’s instructions. The following standard thermal profile was used for all PCRs: 95 °C for 2 min; 40 cycles of 95 °C for 10 s and 60 °C for 30 s. Gene expression was normalized to that of *ACTIN2* by subtracting the C_T_ value of *ACTIN2* from the C_T_ value of the gene of interest. Expression ratios were then obtained from the Eq. 2^ΔΔCT^. Primers for genes of interest are listed in Table S1.

### Anthocyanin measurement

Anthocyanin measurement was performed as previous described [54]. The seedlings were grown for 4 days after sowing on 1/2 MS medium, and then used for anthocyanin measurement. Seedlings of each genotype were incubated overnight in 0.6 mL of 1% HCl in methanol at 4 °C and extracted using an equal volume of chloroform after the addition of 0.4 mL of water. After centrifugation, the quantity of anthocyanins was determined by spectrophotometric measurement of the aqueous phase (A530-0.25A657) and normalized to the fresh weight of each sample. 3 independent biological samples were used to measure anthocyanin for each genotype.

### Histochemical GUS staining

Histochemical GUS staining of homozygous T_3_ transgenic lines harboring *AtGLK1*::*GUS* fusion gene was done as previous described [55]. At least 5 individual lines were analyzed to give typical results shown here.

### Statistical analysis

All experiments with each group were performed at least in triplicate. Error bar represents ± S.D. (n = 3). The significant differences between control and treatment of the samples or between wild-type and other genotypes were analysed by the Student’s *t* test. Significant differences from control are denoted by one star corresponding to *P* < 0.05.

## Supplementary Information

**Additional file 1.**

## Data Availability

All data generated or analyzed during this study are included in this published article and its supplementary information files.
