# Supplementary material for: The transcription factor AtGLK1 acts upstream of MYBL2 to genetically regulate sucrose-induced anthocyanin biosynthesis in Arabidopsis
Source: BMC Plant Biol. 2021 May 28;21:242. doi: 10.1186/s12870-021-03033-2 (PMC8162001; doi:10.1186/s12870-021-03033-2)
Supplement: Supplementary file 1 — Additional file 1. [file 12870_2021_3033_MOESM1_ESM.doc]

**The transcription factor AtGLK1 acts upstream of MYBL2 to genetically regulate sucrose-induced anthocyanin biosynthesis in Arabidopsis**

Dongming Zhao^1†^, Yuxuan Zheng^1†^, lingjun Yang^1^, Ziyu Yao^1^, Jianfeng Cheng^1^, Fang Zhang^1^, Haiyan Jiang^1^, Dong Liu^1*^

^1^ College of Agronomy/Key Laboratory of Crop Physiology, Ecology and Genetic Breeding, Ministry of Education, Jiangxi Agricultural University, Nanchang 330045, China

^†^ Dongming Zhao and Yuxuan Zheng contributed equally to this work

**^*^** Corresponding author

**Author of correspondence:**

Dong Liu

College of Agronomy/Key Laboratory of Crop Physiology, Ecology and Genetic Breeding, Ministry of Education

Jiangxi Agricultural University

Nanchang 330045

China

Tel: (86) 0791-83828081

Fax: (86) 0791-83828081

E-mail: [liudjxau@126.com](mailto:liudongbio@163.com)

**Supplemantary Data**

**
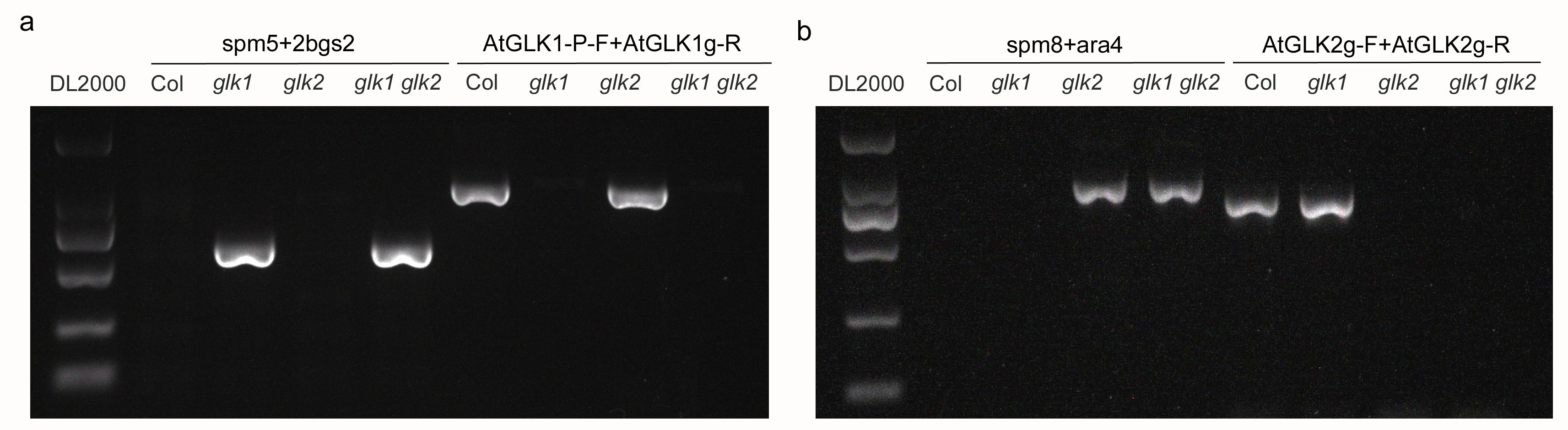
**

**Fig. S1.** PCR-based identification of *dSpm* insertion mutants. DNA was extracted from 4-d-old seedlings and subjected to PCR using specific primers. (**a**) For *AtGLK1*, primers were designed to amplify a 600-bp fragment from the *glk1* mutant (spm5+2bgs2) and a 1111-bp fragment from the wild-type (Col) seedlings (AtGLK1-P-F+AtGLK1g-R). (**b**) For *AtGLK2*, primers were designed to amplify a 885-bp fragment from the *glk2* mutant (spm8+ara4) and a 770-bp fragment from the wild-type (Col) seedlings (AtGLK2g-F+AtGLK2g-R).


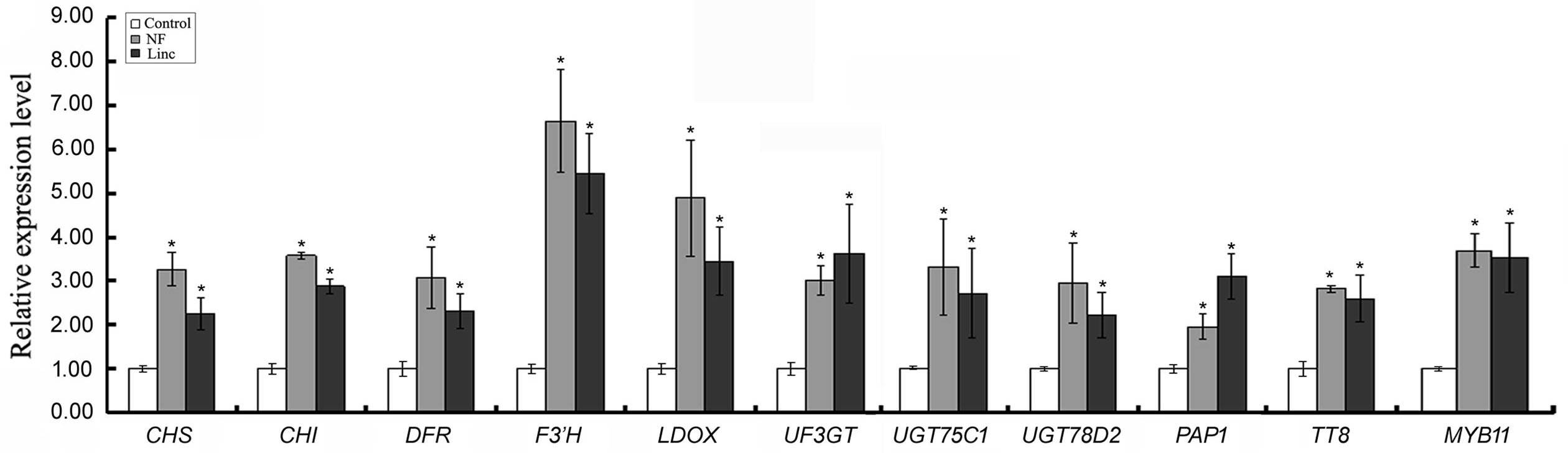


**Fig. S2.** Effect of norflflurazon (NF) and lincomycin (Linc) on the transcript levels of structural (*CHS*, *CHI*, *DFR*, *F3*'*H*, *LDOX*, *UF3GT*, *UGT75C1*, and *UGT75C2*) and regulatory (*PAP1*, *TT8*, and *MYB11*) genes involved in anthocyanin biosynthesis in 4-d-old wild-type seedlings. The asterisks indicate statistically significant differences compared with the corresponding wild-type (Student’s *t* test: **P*＜0.05).


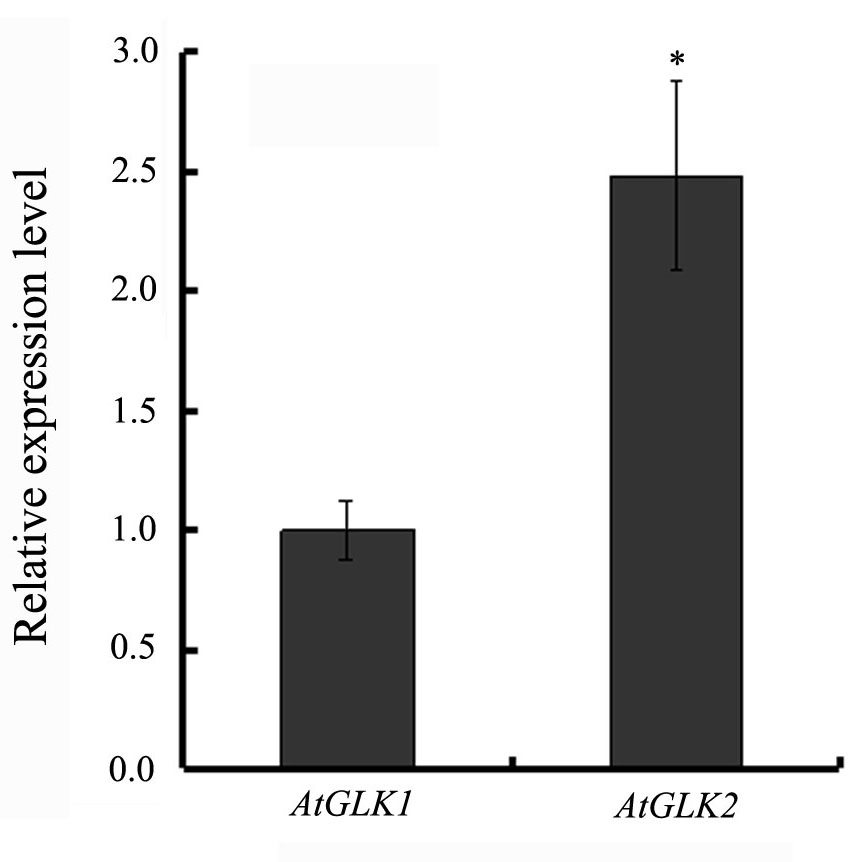


**Fig. S3.** Expression levels of *AtGLK1* and *AtGLK2* in 4-d-old wild-type seedlings. The asterisk indicates statistically significant differences between *AtGLK1* and *AtGLK2* genes (Student’s *t* test: **P*＜0.05).

**Table S1. List of primers used in this study**

| **Primer name** | **Primer sequence (5ˊ-3ˊ)** | **Function** |
| --- | --- | --- |
| *PGLK1-F* | CGCGGATCCTGCCGTACGAATATTGTGAAC | Amplification of *AtGLK1* promoter |
| *PGLK1-R* | CATGCCATGGCGATCAATCTTCACTTGTAG |
| *OEGLK1-F* | GCTCTAGAATGTTAGCTCTGTCTCCG | Amplification of *AtGLK1* coding region |
| *OEGLK1-R* | GCGAGCTCTCAGGCACAAGACGCGGTC |
| *OEMYBL2-F* | CATGCCATGGTAATGAACAAAACCCGCCTTC | Amplification of *MYBL2* coding region |
| *OEMYBL2-R* | CATGGGTAACCTCATCGGAATAGAAGAAGCG |
| *rAtGLK1-F* | TATGACGGTGACAGTGACCGG | Quantitative PCR analysis of *AtGLK1* gene |
| *rAtGLK1-R* | AACTGTTCCACTGCCTCCACG |
| *rAtGLK2-F* | TGTGTGTAAGCAAGAGGGTGG | Quantitative PCR analysis of *AtGLK2* gene |
| *rAtGLK2-R* | CTACCCCTAATTGCTCCACCG |
| *rCHS-F* | GGCAAAGAAGCGGCAGTGAAG | Quantitative PCR analysis of *CHS* gene |
| *rCHS-R* | GGAAGGACGGAGACCAAGAAG |
| *rCHI-F* | TCATGTAGACTCCGTCACGTTTG | Quantitative PCR analysis of *CHI* gene |
| *rCHI-R* | TGACAGATAGAGAAGGAACGGCG |
| *rF3′H-F* | CGGTGGACTGGGCTATAGCTGA | Quantitative PCR analysis of *F3′H* gene |
| *rF3′H-R* | CGAGAGTGGTGTTGGTGGATG |
| *rDFR-F* | CTTTGTTCGTGCCACCGTTCG | Quantitative PCR analysis of *DFR* gene |
| *rDFR-R* | AAAATCCATGGGTGTTGCCAC |
| *rLDOX-F* | GTTTGCAGCTTTTCTACGAGGGC | Quantitative PCR analysis of *LDOX* gene |
| *rLDOX -R* | ATGTTGAGCAAAAGTCCGTGGAG |
| *rUF3GT-F* | TTGTCAGATCGTTTTGGTTCCGC | Quantitative PCR analysis of *UF3GT* gene |
| *rUF3GT-R* | TCTTCCTCACTTTCTCACCGATC |
| *rUGT75C1-F* | GCTGTTTTGGCGCATTGTGC | Quantitative PCR analysis of *UGT75C1* gene |
| *rUGT75C1-R* | TCAGCAAACTGCGGAAACG |
| *rUGT78D2-F* | CGGTGTTGGAGAGTGTATCGG | Quantitative PCR analysis of *UGT78D2* gene |
| *rUGT78D2 -R* | CCAATCTCCCACACAACCTCC |
| *rPAP1-F* | TGGTTCCTGAAGCGACGACAAC | Quantitative PCR analysis of *PAP1* gene |
| *rPAP1-R* | CGCAAACAAATGTTCGAAACAC |
| *rPAP2-F* | CATGAGTCTTCGTGTTGTAAGTC | Quantitative PCR analysis of *PAP2* gene |
| *rPAP2-R* | CTGGCAGACCATTGAGATGGC |
| *rTT8-F* | TGGAGACACCATTGCGTACGT | Quantitative PCR analysis of *TT8* gene |
| *rTT8-R* | TCTTACAAGTACGCGTCCGCT |
| *rMYB11-F* | GATGGCGATTGTAACCCAAGC | Quantitative PCR analysis of *MYB11* gene |
| *rMYB11-R* | ACATGAGGACACGTGGACAGC |
| *rACTIN2-F* | CAAACGAGGGCTGGAACAAGACT | Quantitative PCR analysis of *ACTIN2* gene |
| *rACTIN2-R* | CTGTTGACTACGAGCAGGAGATGG |
| *spm5* | CGGGATCCGACACTCTTTAATTAACTGACACTC | PCR identification of *dSpm* insertion in *glk1* |
| *2bgs2* | AACTGCAGGTTACTGATCCGATTGTTCTT |
| *AtGLK1-P-F* | CTCAATAGGCGGGCCTTATCTAG | PCR identification of *AtGLK1* gene |
| *AtGLK1g-R* | AACTGTTCCACTGCCTCCACG |
| *spm8* | GTTTTGGCCGACACTCCTTACC | PCR identification of *dSpm* insertion in *glk2* |
| *ara4* | TCCGATGTGACCTATATTTC |
| *AtGLK2g-F* | CGACGGAAGACTTGCCGGACTT | PCR identification of *AtGLK2* gene |
| *AtGLK2g-R* | GTGTAACTCCGGCGTCCAATCC |
